# Transcriptome and 16S rRNA analysis revealed the response of largemouth bass (*Micropterus salmoides*) to Rhabdovirus infection

**DOI:** 10.3389/fimmu.2022.973422

**Published:** 2022-10-07

**Authors:** Hui Fei, Shun fa Yi, Hui min Zhang, Yan Cheng, Ya qi Zhang, Xiang Yu, Shi chao Qian, Meng meng Huang, Shun Yang

**Affiliations:** ^1^ College of Life Sciences and Medicine, Zhejiang Sci-Tech University, Hangzhou, China; ^2^ Zhejiang Provincial Key Laboratory of Silkworm Bioreactor and Biomedicine, Zhejiang Sci-Tech University, Hangzhou, China; ^3^ Department of Industrilaztion, Zhejiang Development & Planning Institute, Hangzhou, China; ^4^ Department of Fish disease, Huzhou Baijiayu Biotech Co., Ltd., Huzhou, China

**Keywords:** Micropterus salmoides, rhabdovirus, response, transcriptome, microbiome

## Abstract

To better understand the response of largemouth bass (*Micropterus salmoides*) to *Micropterus salmoides* rhabdovirus (MSRV) infection, we investigated the intestinal bacterial flora and transcriptome profile of fish at 72 hours post-infection (hpi). Total of 1574 differentially expressed genes (DEGs) were identified in largemouth bass spleen following MSRV infection, including 573 upregulated and 1001 downregulated genes. KEGG and GO enrichment analysis revealed that upregulated genes were enriched in certain antiviral related signaling pathway, including NOD-like receptor (NLR), RIG-I like receptors (RLR) and regulation of the interferon (IFN)-γ-mediated signaling pathway, whereas some immune-related DEGs enriched in focal adhesion (FA) and ECM-receptor interaction(ECM-RI) were downregulated, as well as genes associated with metabolic processes, such as peroxisome proliferator-activated receptors (PPAR), adipocytokine signaling pathway, Glycerolipid and Retinol metabolism. Furthermore, the principal component analysis (PCA) and phylogenetic analysis revealed that MSRV infection significantly affected the microbiota of largemouth bass intestine; the LEfSe analysis showed that relative abundances of *Streptococcus* were significantly increased, while the content of *Akkermansia, Enterococcus* and *Lactobacillus* were remarkably decreased in the fish intestine following MSRV infection. Additionally, a high correlation was determined between the expressions of interferon-related upregulated genes and the relative abundance of *Streptococcus* by redundancy analysis (RDA). These results collectively illustrated that intestinal microbiota composition might be associated with the immune-related gene expression in largemouth bass in response to MSRV infection.

## Introduction

The largemouth bass (*Micropterus salmoides*) is an economic freshwater fish species in aquaculture owing to its high preference by consumers worldwide (1, 2). In decades, frequent outbreaks of disease have affected the largemouth bass farming (3, 4). Among these diseases, MSRV causes serious diseases occurring in juvenile fish, leading to huge economic losses (5, 6). Therefore, it is urgent and necessary to detect an effective way to prevent and treat this rhabdovirus disease in juvenile largemouth bass, which also requires us to deeply study the interaction between MSRV and fish.

In the last decades, transcriptome sequencing was applied to understand how did the virus infection affect physiological processes of aquatic animals (7–11). Previous report has demonstrated that megalocytivirus-induced gene expression profiles of flounder at the transcriptome level and uncovered a set of key immune genes and pathways closely linked to megalocytivirus infection (7). Also, transcriptome study has revealed a comprehensive view of the immune system in different clinical phenotype of *Epinephelus moara* naturally infected with nervous necrosis virus (NNV) (8). Moreover, Huang et al. investigated the immune response against *Siniperca chuatsi* rhabdovirus (SCRV) infection in *Siniperca chuatsi* with transcriptome analysis, and demonstrated that *S. chuatsi* could experience a relatively strong change to strengthen the immune system and fight against SCRV infection (9). Phattarunda and his team explored the relationship between metabolic gene expression and antiviral responses in *Fenneropenaeus merguiensis* using transcriptome analysis, and stated that fructose-6-phosphate aminotransferase was considered as metabolic genes whose expression was related to white spot syndrome virus (WSSV) infection (10). As for largemouth bass, only one report demonstrated that MSRV infection induced apoptosis and activated interferon signaling pathway in largemouth bass skin cells with transcriptome analysis (11). Nevertheless, transcriptome analysis of the response of largemouth bass to MSRV infection *in vivo* has not been described to our knowledge.

In recent years, interactions of commensal microbiota and viral infection have been studied, demonstrating a third player in the interaction between hosts and viruses (12). Growing evidence have shown that the viral infection can change microbiota composition of the host. For example, Yildiz and colleagues demonstrated an overall reduction of the microbial content in small intestine of mice post influenza infection, but an increase in the relative abundance of *Gammaproteobacteria* and a drop of *Bacteroidia* (13). Similarly, Deriu et al. reported that influenza infection can alter the intestinal microbiota profile, leading to an enrichment of Proteobacteria and depletion of obligate anaerobic bacteria in the gut (14). In aquatic animals, Huang et al. found that White Feces Syndrome Virus (WFSV) infection dramatically decreased microbial content and diversity in shrimp (15). On the other hand, previous studies have demonstrated that intestinal microbiota could affect viral infection in fish (16–19). Galindo-Villegas et al. found that commensal microbes in newly hatched zebrafish (*Danio rerio*) primed neutrophils and induced several genes encoding pro-inflammatory and antiviral mediators, enhancing the resistance of larvae to Spring Viremia of Carp Virus (SVCV) infection, indicating that the intestinal microbiota contributed to the antiviral capability of zebrafish (18). Baldridge’s team stated that the bacterial microbiota limits the efficacy of IFN-λ-dependent innate immunity or alters some yet-undefined innate immune pathway that renders viruses susceptible to the effects of IFN-λ (19). These results evidenced that the interaction between intestinal microbiota and viral infection also existed in aquatic animals.

Regarding the interaction mechanism between the intestinal microbiota and gene expression of host, previous studies have demonstrated that about 10% of the host transcriptome is regulated by microbes, mainly including genes in immune, cell proliferation, and metabolic functions. The influence of microbes on host gene expression is highly site-specific, and each cell fraction is enriched with specific transcriptional regulators (20). For example, it was well demonstrated that gut bacterial communities interact with gene expression, and most of the observed genes were involved in immunology, the bacterial community, and cell differentiation in the Huanghe carp new strain (21). However, little information is currently available on the interaction between the intestinal microbiota and immune related gene expression of largemouth bass during MSRV infection.

In present study, the host gene expression and the intestinal microbiota diversity of largemouth bass following MSRV infection were analyzed using transcriptome profiling and 16S rRNA gene sequencing, respectively. Furthermore, the relationship between intestinal microbiota and transcriptome profile of largemouth bass was analyzed. This project aimed to better understand the response of largemouth bass to MSRV infection, and clarify the relationship between intestinal microbiota and immune-related genes expression of largemouth bass during MSRV infection.

## Materials and methods

### Fish

Healthy largemouth bass (“Zhelu No 1”) was obtained from Huzhou Baijiayu Biotech Co., Ltd. (Huzhou, China). The control and infection groups (300 specimens each, average body length 4.15cm) were reared in two separate tanks at 25 ± 1°C with commercial feed for one week before investigation. Fish were anesthetized using MS-222 before sacrifice and sampling.

### Infection and sampling

MSRV was isolated from diseased largemouth bass in Huzhou, and propagated in Epithelioma Papulosum Cyprini (EPC) cells. The stocks of MSRV were stored at -80°C before use. For infection, the concentration of MSRV was adjusted to 5.0×10^4^ TCID_50_/mL, and then fish was intraperitoneally injected with 10 µL of MSRV using 50 µL volume micro-syringe. The fish in the control group was intraperitoneally injected with 10 µL PBS (pH 7.4, 50mM).

The result pre-experiment for MSRV infection showed that the death of largemouth bass occurred at 72h following MSRV infection, and the mortality was calculated to be 60% during 14 days of observation (**Figure S1**). Therefore, three individual spleen samples from the control and infection group were randomly sampled from alive fish at 72 h post-infection for transcriptome sequencing analysis. Each individual sample contains eighteen alive fish. The whole intestines sample from the same fish (as used for the transcriptome analysis) was used for 16S RNA sequencing. All tissues were frozen in liquid nitrogen and stored at -80 °C until use.

### RNA extraction, cDNA library construction, sequencing, *de novo* assembly and annotation

Total RNA was extracted from spleens from the fish in the control and infection group for cDNA library construction as described before (22, 23). Then, the qualified library was pooled based on pre-designed target data volume and then sequenced on Illumina sequencing platform at Beijing Biomarker Technologies Co., Ltd. (Beijing, China). *De novo* assembly and annotation were also manipulated according to our previous study (24, 25). Briefly, Q20, Q30, GC-content and sequence duplication levels of the clean data were calculated. Genes were annotated by querying against GO (26) and KEGG (27) databases using BLASTx (28).

### Differential expression analysis and functional enrichment

The expression level of each transcript was calculated according to the reads per kilobase of exon per million mapped reads (RPKM) method (21). Then, the differential expressed genes (DEGs) in control and infection groups were identified using the DESeq2. Genes with expression fold change ≥ 2 and FDR < 0.01 found by DESeq2 were assigned as differentially expressed genes. Moreover, GO enrichment analysis was carried out with GOATOOLS (https://github.com/tanghaibao/Goatools) for analysis of DEGs. KEGG pathway enrichment analysis was operated with KOBAS (http://kobas.cbi.pku.edu.cn/home.do) at a Bonferroni-corrected *p*-value ≤ 0.05 compared with the whole-transcriptome background.

### Quantitative real-time PCR

To verify the reliability of the DEG data, nine representative DEGs were randomly chosen for qRT-PCR analysis. Briefly, RNA was extracted from the collected spleens, then synthesized cDNA after quality evaluation using a Nanodrop 2000. Subsequently, the cDNA was adjusted 100 ng/μl, and qRT-PCR was performed with SYBR Green I Master Mix (Tiangen, China) in a LightCycler^®^ 480 II real time system (Roche, Switzerland). Each assay was conducted in triplicate with the *β-actin* gene as the internal reference, and analyzed relative to the *β-actin* gene by the 2^-ΔΔCt^ method (29, 30). In addition, six DEGs involved in interferon related genes and RIG-I-like receptor genes were also analyzed by qRT-PCR as above. All primers for the qRT-PCR were designed based on specific sequences and listed in **Table S1**.

### High throughput sequencing of the 16S rRNA gene

The intestinal contents were collected from whole intestines of juvenile fish in the control and infection groups for high-throughput sequencing of the 16S rRNA gene. The microbial DNA was extracted using the E.Z.N.A.^®^ Soil DNA kit (Omega Biotek, Norcross, GA, U.S.). The V3-V4 region of the bacterial 16S ribosomal RNA gene was amplified by PCR using the primers 338F 5’-ACTCCTACGGGAGGCAGCAG-3’; 806R 5-GGACTACHVGG-TWTTAAT-3’. Then, amplicon DNA was extracted and purified, and SMRTbell libraries were prepared by blunt-end ligation according to the manufacturer’s instructions (Pacific Biosciences). Subsequently, purified SMRTbell libraries were sequenced by Beijing Biomarker Technologies Co., Ltd. (Beijing, China).

According to the quality of single nucleotide, raw data was primarily filtered by Trimmomatic (31) (version 0.33). Identification and removal of primer sequences was processed by Cutadapt (32) (version 1.9.1). PE reads obtained from previous steps were assembled by USEARCH (33) (version 10) and followed by chimera removal using UCHIME (34) (version 8.1). The high-quality reads generated from the above steps were used in the following analysis. Then, OTU clustering, species annotation, Alpha, Beta-diversity, microbial community bar plots, heat maps, phylogenetic analyses, PCA (principal component analysis), LEfSe (linear discriminant analysis effect size) and RDA were performed using BMKCloud (www.biocloud.net).

### Statistical analysis

Statistical analysis was performed using SPSS software (Version 20.0; SPSS, Inc). Significant differences in data were analyzed by one-way ANOVA with Duncan’s multiple range test (DMRT). The *p*-value less than 0.05 (*P < 0.05*) in these analyses indicated significant difference.

## Results

### Differential gene expression between the infection and control group

**Figure 1:**
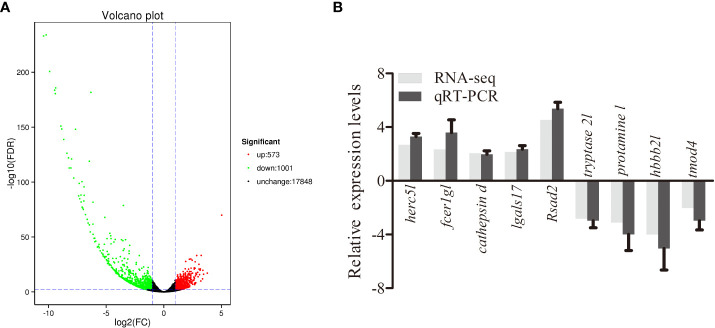
Analysis and validation of DEGs. **(A)** The “volcano plot” picture of DEGs following MSRV infection. Red spot, up-regulated; green spot, down-regulated; black spot, no difference in expression. **(B)** Validation of RNA-seq data by qRT-PCR analysis. X-axis, gene name; Y-axis, fold change in gene expression. The relative expression of 10 DEGs were determined by qRT-PCR and compared with the results of RNA-seq.

To analyze the response of largemouth bass to MSRV infection in gene levels, spleens were collected at 72 hpi for transcriptome analysis. After sequencing, an average of 5.74Gb clean reads was obtained from 36.62Gb raw reads for all samples. The GC content was 48.29% and Q30 was 93.83% (**Table S2**). The average mapping rate for all clean reads ranged from 94.17% to 95.04% (**Table S3**). Then, the DEGs were identified on the basis of expression fold change ≥ 2 and FDR < 0.01. A total of 1574 genes were differentially expressed in the spleen following MSRV infection, including 573 upregulated and 1001 downregulated genes (**Figure 1A**).

**Figure 2:**
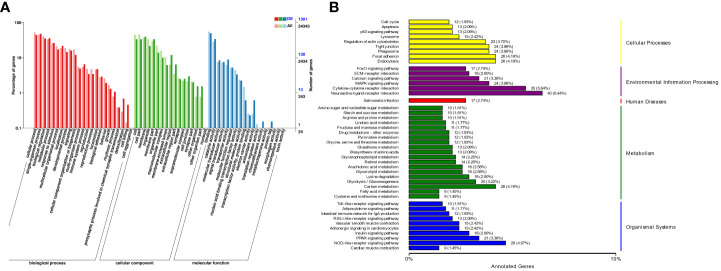
The Cluster analysis of DEGs in transcriptome following MSRV infection. **(A)** The GO functional classification of DEGs. **(B)** KEGG functional classification of DEGs.

**Figure 3:**
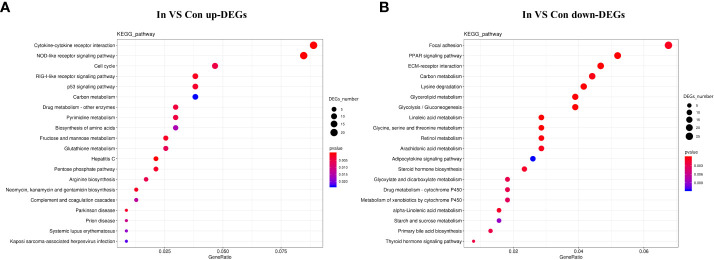
KEGG enrichment analysis of DEGs in transcriptome following MSRV infection. **(A)** KEGG enrichment analysis of up-regulated genes following MSRV infection. **(B)** KEGG enrichment analysis of down-regulated genes following MSRV infection.

The enrichment of DEGs after MSRV infection was analyzed using Encyclopedia of GO and KEGG bases for functional analysis. By GO annotation, genes were classified into the following functional categories: 23 terms in the biological process, 17 terms in the cellular component, and 18 terms in the molecular function categories (**Figure 2A**). The KEGG analysis showed that the DEGs were annotated in many immune related pathways, including FA, Endocytosis, Neuroactive ligand-receptor interaction, NLR signaling pathway and Cytokine-cytokine receptor interaction (**Figure 2B**). Furthermore, KEGG enrichment analysis showed that many upregulated genes were enriched in NLR and RLR signaling pathway (**Figure 3A**). While, immune-related DEGs enriched in FA and ECM-RI were downregulated, and other downregulated genes were associated with metabolic processes, such as PPAR, adipocytokine signaling pathway, Glycerolipid and Retinol metabolism, based on the KEGG database (**Figure 3B**). The GO enrichment analysis also showed that many upregulated genes were enriched in immune response and regulation of the IFN-γ-mediated signaling pathway (**Figure S2**). Moreover, Further analysis showed that MSRV infection could induce upregulated expression of interferon-related genes and RLR signaling pathway that play an important role in antiviral response (**Table 1**).

**Table 1:** Partial of DEGs involved in Interferon related gene and RIG-I-like receptor signaling pathway.

Gene name	Gene ID in transcriptom	Log2FC	Regulated	FDR
**Interferon related gene**				
*IFN I c*	Micropterus_salmoides_newGene_5155	2.67101	up	2.28E-07
*IFN γ-L*	gene-LOC119894803	1.96589	up	0.000134
*IFNaR2-L*	gene-LOC119903524	1.72337	up	3.93E-17
*TRIM16-L*	gene-LOC119897002	1.81832	up	4.93E-05
*TRIM39-L*	gene-LOC119912470	2.62994	up	7.23E-14
*HERC5*	gene-LOC119882585	1.42287	up	5.85E-17
*IFIT5-L*	gene-LOC119915782	1.03691	up	0.000615
*IFIT5-L*	gene-LOC119915783	1.01904	up	0.000587
*IFI27-L2a*	gene-LOC119910835	1.81691	up	0.000812
*IFI27-L2b*	gene-LOC119910755	1.96394	up	9.25E-13
*Polyubiquitin b-L*	gene-LOC119891167	1.89023	up	5.64E-17
*Polyubiquitin b-L*	gene-LOC119891171	1.85923	up	1.02E-21
**RIG-I-like receptor signaling pathway**				
*TRIM16-L*	Micropterus_salmoides_newGene_1464	1.70092	up	8.78E-06
*TRIM16-L*	Micropterus_salmoides_newGene_1465	1.87486	up	1.97E-07
*Permeability F2-L*	gene-LOC119887768	1.572511	down	8.17E-08
*Permeability F2-L*	gene-LOC119899104	1.09787	up	0.000211
*IL8-L*	gene-LOC119899106	1.376985	down	0.001496
*TRIM25-L*	gene-LOC119899732	1.17277	up	0.000154
*Caspase 8-L*	gene-LOC119902448	1.04332	up	6.86E-06
*HP F2P81*	gene-LOC119903833	1.15587	up	0.001073
*Permeability F2-L*	gene-LOC119912333	1.988367	down	5.44E-05
*CP Ras 1-L*	gene-LOC119916009	1.037312	down	0.001215
*CXCL 19*	gene-cxcl19	1.14246	up	0.000128
*DHX 58*	gene-dhx58	1.19961	up	3.55E-16

### Validation of Selected DEGs by RT-qPCR

**Figure 4:**
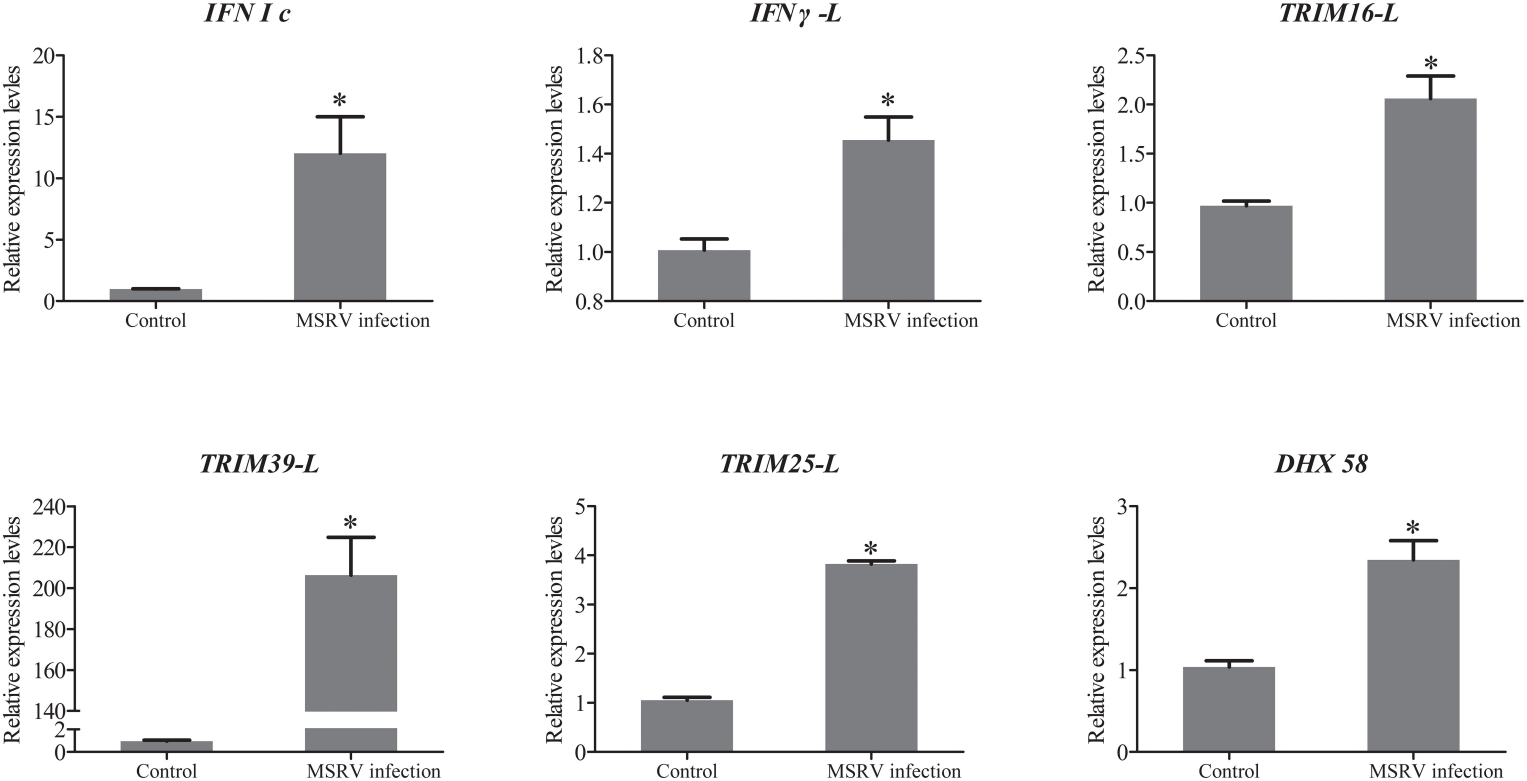
The qRT-PCR analysis of expression of genes involved in Interferon related genes and RIG-I-like receptor genes following MSRV infection. Asterisk represented statistical significance between infection group and control group. (*p* < 0.05).

To further evaluate our DEG library, the mRNAs of 5 upregulated DEGs and 4 down-regulated DEGs were randomly selected to be measured by qRT-PCR. As shown in **Figure 1B**, the qRT-PCR results showed similar expression tendency as the high-throughput sequencing data, despite some quantitative differences at the expression level. Moreover, qRT-PCR analysis showed that six genes involved in interferon related genes and RIG-I-like receptor genes, including *IFN I c, IFN γ-L, TRIM16-L, TRIM39-L, TRIM25-L* and *DHX 58*, were up-regulated following MSRV infection, which also showed similar expression tendency to high-throughput sequencing data (**Figure 4**). The qRT-PCR analysis confirmed the expressions of DEGs detected by the high throughput sequencing analysis.

### Intestinal microbiota of infection and control group

**Figure 5:**
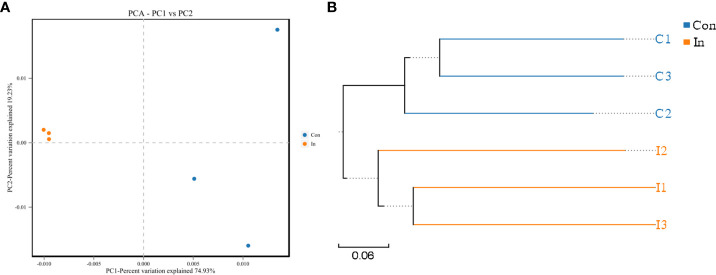
The PCA and phylogenetic analyses of intestinal microbiota of largemouth bass following MSRV infection. **(A)** PCA plot of intestinal bacterial communities between the infection and control group. **(B)** UPGMA tree based on weighted UniFrac distances. The Con means control group; In means infection group.

Nested PCR analysis showed that MSRV was detected in gut intestinal contents following virus infection (**Figure S3**). Subsequently, the same fish (as used for the transcriptome analysis) were used to 16S rRNA sequences and identify the intestinal microbiota diversity of largemouth bass. After sequencing, a total of 479630 raw reads and 478618 clean reads were obtained from 12 samples. For all valid tags, Q30 accounted for 97.20% and GC content was 54.37% (**Table S4**). To better understand the bacterial diversity difference of microbial communities in these two groups, the number of observed species, along with the Shannon, Simpson, and Chao1 indices, were calculated from the OTUs (**Table 2**). PCA was performed to reflect the differences and distances between microbes among control and infection groups. The results showed that intestinal microbiota of the infection group was grouped separately, and a 74.93% distance in PC1 was observed (**Figure 5A**). MSRV infection could influence the recombination process of the intestinal microbial community. Moreover, the results of the multiple sample similarity tree showed that the intestinal microbiota of the control group was very different from those of the infection group (**Figure 5B**). The PCA and phylogenetic analyses divided all intestine samples into two parts: control and infection.

**Table 2:** Alpha diversity of intestinal microbiota of largemouth bass following MSRV infection.

Groups	ACE	Shannon	Simpson	Chao1
Con	804.67 ± 72.40	6.71 ± 0.36	0.96 ± 0.0104	899.96 ± 93.45
In	609.67 ± 5.86*	8.22 ± 0.05*	0.99 ± 0.0005*	844.37 ± 106.72

Con means control group; In means infection group. Asterisk represented statistical significance between infection group and control group. (p < 0.05).

**Figure 6:**
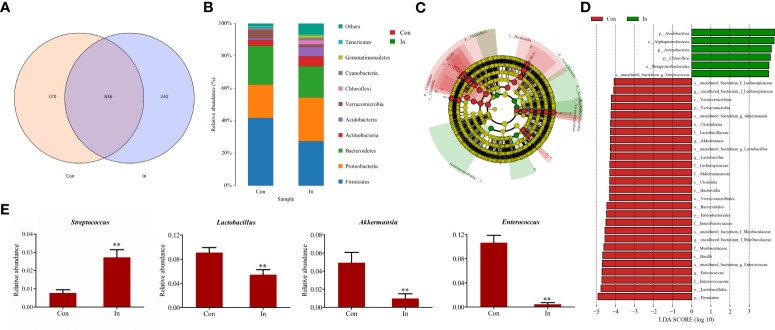
Difference analysis of intestinal microbiota of largemouth bass following MSRV infection. **(A)** The Venn diagram for showing OUT numbers. **(B)** The bar plots for Microbial community. **(C)** Lefse analysis of differential flora. LDA threshold is 4.0; red node indicates enrichment in the control group; green node indicates enrichment in the infection group. **(D)** The LDA scores of different microbes between control group and infection group. Red means the control group; green means the infection group. **(E)** Comparison of the relative abundance of partial different microbes between control group and infection group. Two asterisk represented statistical significance between infection group and control group. (*p* < 0.01).

Furthermore, the Venn diagram showed that the number of intestinal microbial OTU in the control group was more than that in the infection group (**Figure 6A**). Microbial community bar plots at phylum level showed that Firmicutes was the dominant taxon in the infection group, followed by Bacteroidetes and Proteobacteria; while in the control group, Firmicutes was the dominant taxon, followed by Proteobacteria and then Bacteroidetes (**Figure 6B**). The Firmicutes/Bacteroidetes ratio fell from 1.74 (Control group) to 1.43 (Infection group). In addition, through the LEfSe analysis, we found the relative abundance of *Streptococcus* in the intestinal tract of infection group was higher than that of the control group. At the same time, the *Akkermansia*, *Lactobacillus* and *Enterococcus* content was lower than that of the control group at genus level (**Figures 6C–E**).

### Differentially expressed genes related to the intestinal microbiota

**Figure 7:**
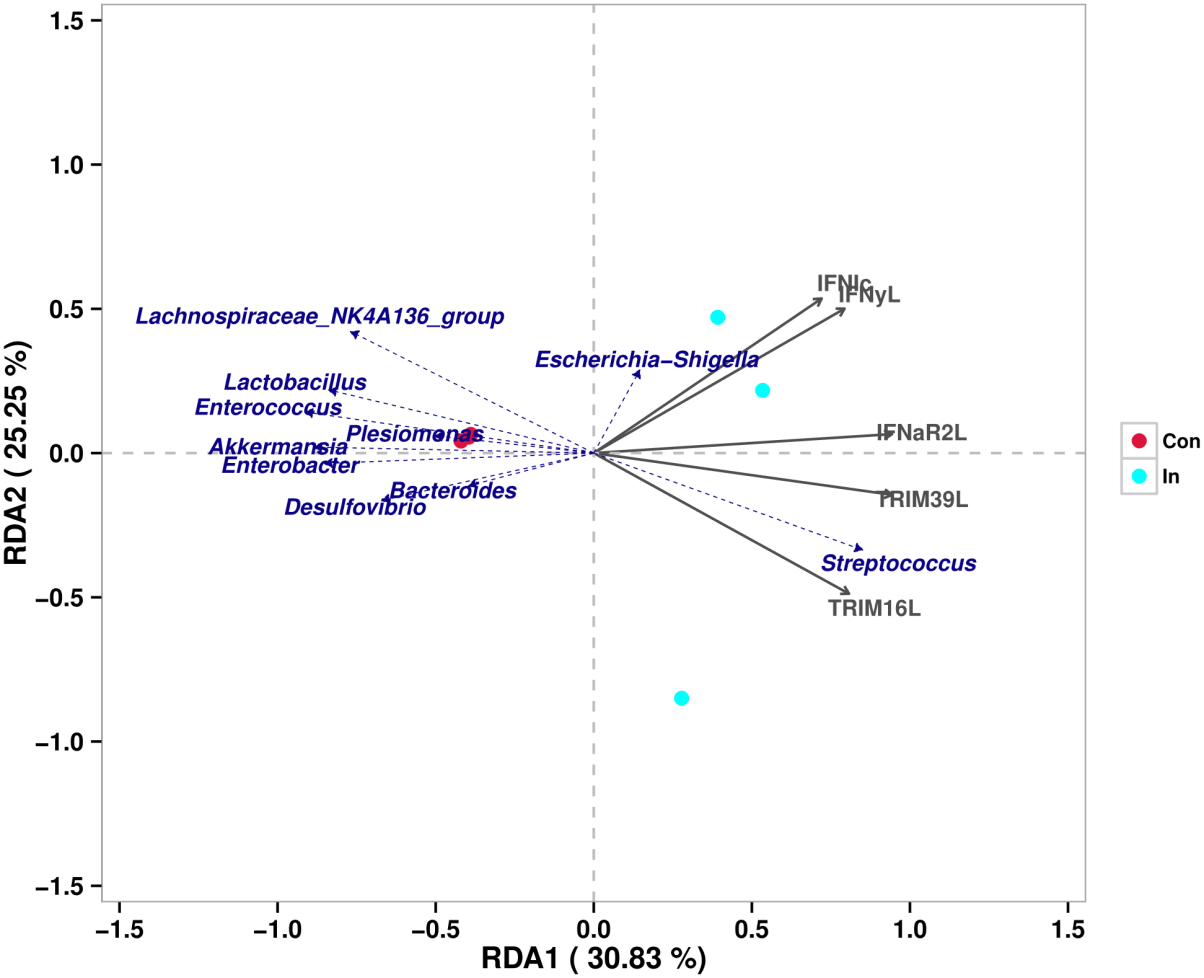
Redundancy analysis of the correlation between different intestinal microbes and interferon-related differentially expressed genes.

RDA was used to infer the relationship between the composition of the intestinal microbiota on the genus level and DEGs. The expression (Reads per kilobase million, RPKM) of partially investigated interferon-related genes (*IFN I C, IFN γ-L, IFNaR2-L, TRIM16-L, TRIM39-L*) were used as the environmental factors in the RDA analysis. The results revealed a high correlation existed between the relative abundance of *Streptococcus* and the expressions of these interferon-related genes (**Figure 7**).

## Discussion

To study the response of largemouth bass to MSRV, high-throughput transcriptomic analysis was used to clarify the gene expression profiles after MSRV infection *in vivo* in present study. Previous report revealed that over 60% juvenile largemouth bass died after intraperitoneal injection of MSRV for 48 h (35) and 80% fish died at 5 days post-infection(dpi), no more death occurred until the end of 14 days challenge experiment. While, another report stated that almost 30% of largemouth bass died after intraperitoneal injection of MSRV for 48 h, and all died at 8 dpi (36). In our study, death of fish (“Zhelu No 1”) occurred at 72 h following MSRV infection, and 60% fish died at 9 dpi. These discrepancies may be attributable to the virulence difference of MSRV as well as the difference in quality of fish germplasm. Additionally, the latest report revealed that the viral load of largemouth bass (“Zhelu No 1”) reached a plateau at 48-72 hpi in spleen and kidney during MSRV infection (37). Taken together, the sampling time at 72 hpi for transcriptome sequencing analysis was reasonable in current study.

Considering the immune related gene expression patterns of fish after virus infection, previous study illustrated that IκBα-like protein A*(IκBαLA)* coding gene was down-regulated in RLR, mitogen-activated protein kinase (MAPK), Toll-like receptor (TLR), T cell receptor (TCR), B cell receptor (BCR), NF-κB signal pathways in grass carp (*Ctenopharyngodon idella*) after Grass Carp Reovirus (GCRV) infection (38). Besides the influence on *in vivo*, GCRV infection also induced the up-regulation of immune-related genes, including *viperin*, antigen peptide transporter 1 (*tap1*), toll-like receptor 3 (*tlr3*), and interferon regulatory factor-1 (*irf11*) in *Ctenopharyngodon idellus* kidney (CIK) cells A (39). Considering the MSRV infection, it was well demonstrated that molecules in RLR, NLR and nuclear factor-κB (NFκB) pathway were involved in LBS (largemouth bass skin) cells in response to MSRV infection, and suggested that interferon signaling pathway was crucial for MSRV replication like other fish viruses (11, 40–42). Similarly, the transcriptomic analysis in current report revealed that molecules in RLR, NLR and P53 signaling pathway were involved in MSRV infection. For example, interferon-related genes, including IFN I c, IFN γ-L, IFNaR2-L, TRIM16-L and TRIM39-L were up-regulated; RLR-related genes, including TRIM16-L, Permeability F2-L, TRIM25-L and Caspase 8-L were also up-regulated after MSRV infection. These results suggested that RLR signaling pathway was activated after MSRV infection, which may regulate the antiviral response mediated by interferon. Nonetheless, immune-related DEGs that enriched in FA and ECM-RI were downregulated during MRVS infection. Previous report revealed that inhibition of FA kinase signaling leads to reduced viral replication at later times of influenza A infection in normal human bronchial epithelial (NHBE) cells (42). The DEGs related to ECM-RI in grass carp was down-regulated during the middle (24h) and late (72h) ages of GCRV infection (38). Consistent with the above results, our experimental data also showed the DEGs related to ECM-RI and FA were downregulated after MSRV infection at 72hpi. It is speculated that genes involved in ECM-RI and FA might be suppressed at later times of MSRV infection, and thus led to reduced viral replication.

Regarding the impact of virus infection on other physiological functions, published report revealed that infectious hypodermal and haematopoietic necrosis virus (IHHNV) infection could decrease the expression of a large amount of genes related to metabolic function, and further might affect the growth of *Procambarus clarkia* (43). Chen et al. demonstrated that DEGs in grass carp were mainly enriched in lipid metabolism during the middle and late ages of GCRV infection (39). In according with this, many DEGs associated with lipid metabolism, such as PPAR, adipocytokine and cytochrome P450 signaling pathway of largemouth bass were also downregulated at late ages of MSRV infection in current study. The adipocytokine signaling pathway may be the central signaling pathway in eating disorder. which induces abnormal body weight (44). The PPAR signaling pathway is an important regulatory pathway involved in lipid metabolism, adipocyte proliferation and differentiation, which plays a significant role in the metabolism and growth rate of black porgy (45). The cytochrome P450 enzyme, a conserved group of proteins that serve as key players in the metabolism of organic substances and the biosynthesis of important steroids, lipids, and vitamins in eukaryotes (46). Thus, we speculated that MSRV infection might changes the permeability of the cell membrane by affecting the structure of lipids in the cell membrane, leading to cell death of largemouth bass, which should be validated in further research.

To better understand the intestinal microbiota structure in response to MSRV infection, the differences of intestine bacterial community structures in the control and infection groups were explored. In many cases, viral infection can change microbiota composition of the host (13–16, 47, 48). In this study, the decreased OTU number in infection group indicated that MSRV infection could cause component alterations in intestinal microbes, and reduce the diversity of intestinal flora. Kang and Cai stated that human immunodeficiency virus (HIV) infection significantly increased the abundance of Firmicutes and Proteobacteria, and decreased Bacteroidetes content (48). Consistently, the abundance of Bacteroidetes was also decreased in the present study. In addition, the relationship between Firmicutes and Bacteroidetes, expressed as the Firmicutes/Bacteroidetes ratio, has been associated with several pathological conditions (49). In our study, the Firmicutes/Bacteroidetes ratio fell from 1.74 (Control) to 1.43(Infection), which indicated that MSRV infection could indeed affect the intestinal flora of largemouth bass. Furthermore, through LEfSe analysis, we found a higher abundance of *Streptococcus* in the infection group. *Streptococcus*, including *Streptococcus iniae* and *S. agalactiae* is an important emergent pathogen that affects many fish species worldwide, especially in warm-water regions (50, 51). Interestingly, previous study demonstrated that intranasal influenza virus infection enhances host cell- group A *Streptococcus* (GAS) adhesion and invasion, which may be caused by GAS directly binds to virus-infected cells and viral particles (52). More recently, a landmark study by Bai and his team revealed that influenza-A-virus-induced Cyclophilin A interacts with focal adhesion kinase (FAK) and inhibits cCbl-mediated, K48-linked FAK ubiquitination, which promotes GAS coinfection *via* the FAK/Akt pathway (53). This suggested that MSRV infection may also somehow affect the action of *Streptococcus* through a series of immune regulatory pathways of the host, and further lead to superinfection. In addition, the relative abundance of *Akkermansia, Enterococcus* and *Lactobacillus* decreased following MSRV infection. Growing evidences have demonstrated the probiotic effect of *Akkermansia, Enterococcus* and *Lactobacillus* in the gut of host (54–56), in which *Akkermansia*, and *Lactobacillus* were suggested to afford anti-inflammatory action in colitis (56, 57), while *Enterococcus* had a profound effect on mucosa-adherent *Escherichia coli* in the intestine of host (58). In many cases, the abundance of *Akkermansia, Enterococcus* and *Lactobacillus* fluctuated irregularly during different viral infections. For example, H7N9 infection could promote the growth of *Akkermansia* (59), as well as increase the abundance of *Enterococcus faecium* (60). On the contrary, it was observed that *Akkermansia*, significantly decreased in rAAV8- Hepatitis B (HBV)-infected mice (61). As for *Lactobacillus*, its content in intestine decreased after HBV infection (62), while *Lactobacillus* spp. levels were remarkably increased by murine norovirus inoculation during retinoic acid administration (63). This discrepancy may be attributable to intestinal bacteria specificity as well as virus specificity, showing the complexity in mechanisms underlying the intestinal bacteria-mediated antiviral effect.

Generally, Host antiviral immunity is closely correlated with the microbiota composition through poorly understood bi-directional links (64). Gene expression may be a potential mediator of these links between microbial communities and host physiological performance. Richards et al. identified over 5,000 host genes that change expression, including 588 distinct associations between specific taxa and host genes, and demonstrated that specific microbes play an important role in regulating the expression of individual host genes (65). Similarly, it was well evidenced that 15 microbial families were all highly correlated with host expression of immune genes related to macrophage and B cell functions in Stickleback, and vice versa (66). For example, previous study demonstrated significant associations between host genetic variation and microbiome composition in 10 of the 15 body sites tested. These associations are driven by host genetic variation in immunity-related pathways, and are especially enriched in host genes that have been previously associated with microbiome-related complex diseases (67). As for the present study, after analyzing the intestinal bacterial composition and DEGs in MSRV infection group, we found a high correlation existed between the relative abundance of *Streptococcus* and the expressions of selected interferon-related genes, including *IFN I C, IFN γ-L, IFNaR2-L, TRIM16-L, TRIM39-L*. Previous reports have evidenced that type I interferon production induced by *Streptococcus pyogenes*-derived nucleic acids is required for host protection (68). This suggested that an increase in the abundance of *Streptococcus* might play a role in regulating the expression of interferon-related genes, and lead to activation of the RLR signaling pathway, which need further investigation.

## Conclusion

This study analyzed the gene expression and intestinal microbiota composition of largemouth bass in response to MSRV infection. The results illustrated that some immune-related signaling pathway, including RLP, NLR was regulated, and the abundance of some bacteria, such as *Streptococcus*, *Akkermansia*, *Lactobacillus* and *Enterococcus* was changed in the intestinal tract after MSRV infection. Furthermore, redundancy analysis showed a high correlation existed between *Streptococcus* abundance and the interferon-related genes expression, which suggested that these bacteria might play a role in MSRV infection.

## Data availability statement

The datasets presented in this study can be found in online repositories. The names of the repository/repositories and accession number(s) can be found below: https://www.ncbi.nlm.nih.gov/, SRR18749554 https://www.ncbi.nlm.nih.gov/, SRR18749555 https://www.ncbi.nlm.nih.gov/, SRR18749556 https://www.ncbi.nlm.nih.gov/, SRR18749557 https://www.ncbi.nlm.nih.gov/, SRR18749558 https://www.ncbi.nlm.nih.gov/, SRR18749559 https://www.ncbi.nlm.nih.gov/, SRR18762170 https://www.ncbi.nlm.nih.gov/, SRR18762171 https://www.ncbi.nlm.nih.gov/, SRR18762172 https://www.ncbi.nlm.nih.gov/, SRR18762173 https://www.ncbi.nlm.nih.gov/, SRR18762174 https://www.ncbi.nlm.nih.gov/, SRR18762175.

## Ethics statement

All experimental protocols were conducted in compliance with the relevant provisions for welfare ethics and the protection of experimental animals in the state and Zhejiang Province.

## Author contributions

SYa designed this study. HF conducted almost all experiments and wrote this manuscript. SYi, YC, YZ, XY, SQ and MH participated in the collect of sample, helped, and analyzed the experiments and data. All authors contributed to the article and approved the submitted version.

## Funding

This study was funded by National Natural Science Foundation of China (No. 32102824), the Zhejiang Sci-Tech University Foundation (11612832611909).

## Conflict of interest

Author SQ is employed by Huzhou Baijiayu Biotech Company.

The remaining authors declare that the research was conducted in the absence of any commercial or financial relationships that could be construed as a potential conflict of interest.

## Publisher’s note

All claims expressed in this article are solely those of the authors and do not necessarily represent those of their affiliated organizations, or those of the publisher, the editors and the reviewers. Any product that may be evaluated in this article, or claim that may be made by its manufacturer, is not guaranteed or endorsed by the publisher.
