# Supplementary material for: Transcriptome and 16S rRNA analysis revealed the response of largemouth bass (Micropterus salmoides) to Rhabdovirus infection
Source: Front Immunol. 2022 Oct 7;13:973422. doi: 10.3389/fimmu.2022.973422 (PMC9585208; doi:10.3389/fimmu.2022.973422)
Supplement: Supplementary file 1 [file DataSheet_1.docx]

**Supplementary Material**

**Transcriptome and 16S rRNA analysis revealed the response of largemouth bass (*Micropterus salmoides*) to Rhabdovirus infection**

**Contents of supplementary Material**

**Supplementary Materials and Methods**

Nested PCR

The intestinal contents were collected from whole intestines of juvenile fish in the control and infection groups. Then, RNA was extracted from the collected intestinal contents and cDNA was synthesized. Subsequently, the Nested PCR was performed to amplify the partial sequence of MSRV nucleoprotein gene. Briefly, the PCR was conducted with first pair of specific primers (MSRV-N-F1: CCTGTTGTTGGGGATCTATCG; MSRV-N-R1: GGCATCTCTCTTTTTGGGCAT T), and then the diluted PCR product was used as template to perform second round PCR with second pair of specific primers (MSRV-N-F2: TAAGGAATCGGAGAGAGCAAA; MSRV-N-R2: TTGTGGCAGAGTAAGGGGA). Finally, the agarose electrophoresis was used to detect the PCR amplification products.

## Supplementary Figure S1-S3 and Table S1- S4

**
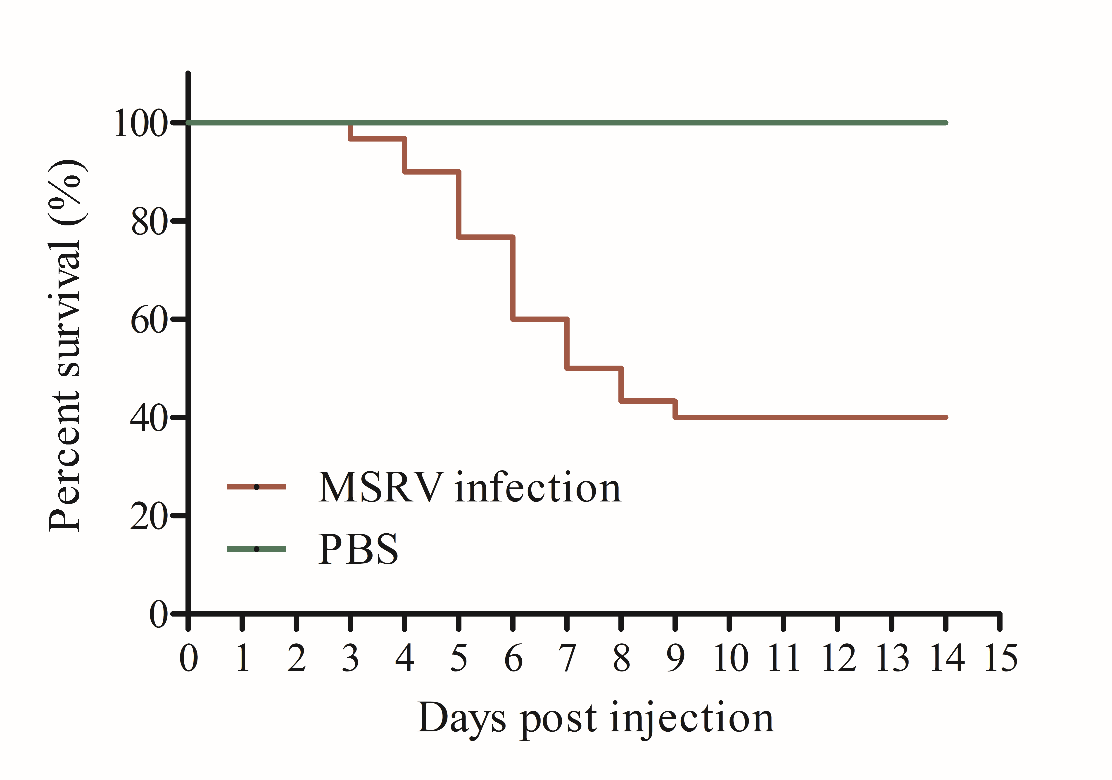
**

**Figure S1** Survival percentage of largemouth bass after MSRV infection.

**
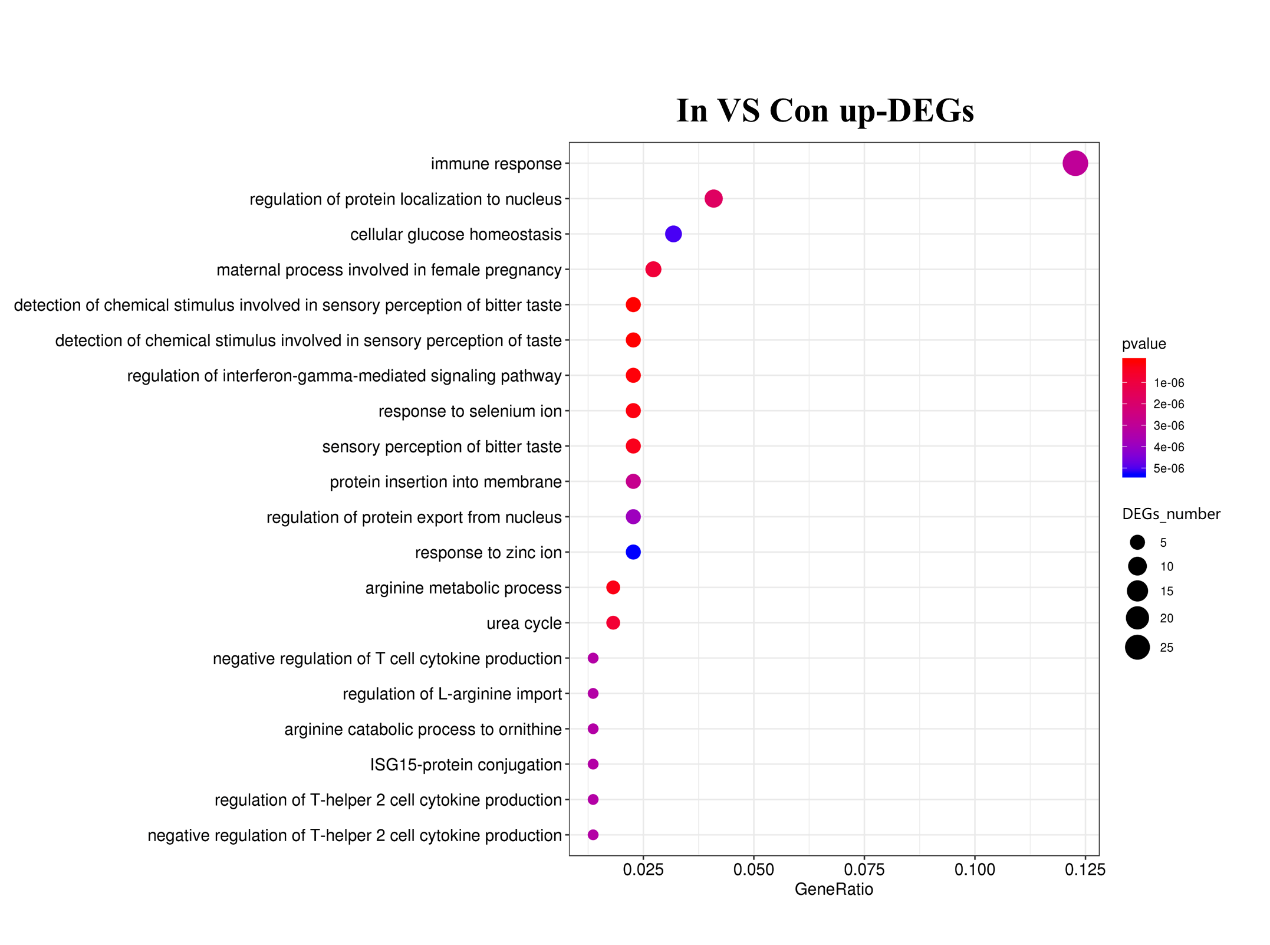
**

**Figure S2.** GO enrichment analysis of up-regulated genes following MSRV infection associated with biological process.

**

**

**Figure S3.** Nested PCR analysis of MSRV in intestinal contents of largemouth bass following viral infection. The nucleoprotein gene of MSRV was detected by nested PCR. Lane M: maker; Lane 1: control group; Lane 2: MSRV infected group.

**TableS1** Sequence information of primers for qRT-PCR.

| **Primer no.** | **Primer name** | **Sequence (5’ - 3’)** | **Gene ID in transcriptom** |
| --- | --- | --- | --- |
| 1 | herc5l-RT-F | CTTCCCTGACACCCTCCGTA | gene-LOC119882585 |
| 2 | herc5l-RT-R | CAGCAGGTATTTCACTCCCG |  |
| 3 | fcer1gl-RT-F | GTTGTGTTATGGCAGAAATCCGT | gene-fcer1gl |
| 4 | fcer1gl-RT-F | TTTTACAGCGTGTGATTGCCC |  |
| 5 | cathepsin d-RT-F | TCTCTGTGGACGGAGTGGCT | gene-LOC119890600 |
| 6 | cathepsin d-RT-R | CACTGTAGTATTTGGGGTCGGTT |  |
| 7 | lgals17-RT-F | GTGAGAGTGGTCTTCCAGGGC | gene-lgals17 |
| 8 | lgals17-RT-R | ACTTTTGGCAGCGGCGTC |  |
| 9 | rsad2-RT-F | ACTCGGTCATCAACACCTTCAAC | gene-rsad2 |
| 10 | rsad2-RT-R | GAACAAGGCAGGGGACGC |  |
| 11 | tryptase 2l-RT-F | CATCCTCAACAGTCAATGGGTG | gene-LOC119896438 |
| 12 | tryptase 2l-RT-R | GTGGGAAGACTCACTGCGGT |  |
| 13 | protamine l-RT-F | GGTCCAAACGAAGGGCACA | gene-LOC119892187 |
| 14 | protamine l-RT-R | CTTGGCGACCTTGCGTTTA |  |
| 15 | hbbb2l-RT-F | TCATCACCAGCATCTTTTCTAACC | gene-LOC119915756 |
| 16 | hbbb2l-RT-R | TCTCGGAGTGGAGGACGCT |  |
| 17 | tmod4-RT-F | AGCACGAGGACAGGGACGA | gene-tmod4 |
| 18 | tmod4-RT-R | TCACTACGCTGTTGATGCCC |  |
| 19 | IFN I c-RT-F | AACACCAACATCTCCTTCCCA | Micropterus_salmoides_newGene_5155 |
| 20 | IFN I c-RT-R | GCCCTCTCCGACAGGTAACTC |  |
| 21 | IFN γ-L-RT-F | GGAGTTGCTTTGGCGTTTG | gene-LOC119894803 |
| 22 | IFN γ-L-RT-R | GTCGTGCTCATTGTGGCTGT |  |
| 23 | TRIM16-L-F | GTCTGGTGTGTCGGGTCTCTT | gene-LOC119897002 |
| 24 | TRIM16-L-R | GACTGTGTCGTGGTCTTTATGGT |  |
| 25 | TRIM39-L-F | CCATAGGAAAGAGGGGTTTAGAT | gene-LOC119912470 |
| 26 | TRIM39-L-R | TTGGAGGGAGGACCAGAGAG |  |
| 27 | TRIM25-L-F | GTCACCAGTCACCACGCTTTC | gene-LOC119899732 |
| 28 | TRIM25-L-R | CCACTTGGCTTATTTCCTCCC |  |
| 29 | dhx58-RT-F | GACTGATGCTTATCGCTCCCTA | gene-dhx58 |
| 30 | dhx58-RT-R | ACGCTTTTACGGGTTTTACTGA |  |
| 31 | β-actin-RT-F | TGGAAGGGACCTCACAGACTAC | MH018565 |
| 32 | β-actin-RT-R | GGGCAACGGAACCTCTCAT |  |

**Table S2** Statistics of clean reads for transcriptome sequencing.

| **Sample ID** | **Clean reads** | **Clean bases** | **GC Content** | **%≥Q30** |
| --- | --- | --- | --- | --- |
| Con1 | 19190023 | 5738093274 | 0.488 | 0.9268 |
| Con2 | 20929922 | 6262462544 | 0.4871 | 0.9362 |
| Con3 | 20581896 | 6157382406 | 0.4798 | 0.9338 |
| In1 | 21402917 | 6395004702 | 0.4835 | 0.9332 |
| In2 | 19553964 | 5843932546 | 0.4822 | 0.954 |
| In3 | 20821171 | 6227216322 | 0.4766 | 0.9456 |

**Abbreviations:** Con means control group; In means infection group.

**Table S3** Statistics of mapped reads for transcriptome sequencing.

| **Sample ID** | **Total Reads** | **Mapped Reads** | **Uniq Mapped Reads** | **Multiple Mapped Reads** | **Reads Map to '+'** | **Reads Map to '-'** |
| --- | --- | --- | --- | --- | --- | --- |
| Con1 | 38,380,046 | 36,240,571 (94.43%) | 33,599,825 (87.55%) | 2,640,746 (6.88%) | 19,841,323 (51.70%) | 19,927,643 (51.92%) |
| Con2 | 41,859,844 | 39,780,878 (95.03%) | 36,833,933 (87.99%) | 2,946,945 (7.04%) | 21,794,292 (52.06%) | 21,877,951 (52.26%) |
| Con3 | 41,163,792 | 38,762,936 (94.17%) | 35,887,561 (87.18%) | 2,875,375 (6.99%) | 21,223,572 (51.56%) | 21,288,035 (51.72%) |
| In1 | 42,805,834 | 40,485,543 (94.58%) | 37,136,852 (86.76%) | 3,348,691 (7.82%) | 22,524,383 (52.62%) | 22,595,981 (52.79%) |
| In2 | 39,107,928 | 37,016,453 (94.65%) | 33,958,429 (86.83%) | 3,058,024 (7.82%) | 20,526,226 (52.49%) | 20,586,214 (52.64%) |
| In3 | 41,642,342 | 39,577,396 (95.04%) | 36,466,664 (87.57%) | 3,110,732 (7.47%) | 21,828,181 (52.42%) | 21,890,111 (52.57%) |

**Abbreviations:** Con means control group; In means infection group.

**Table S4** Statistics for high throughput sequencing of the 16S rRNA gene.

| **Sample ID** | **Raw Reads** | **Clean Reads** | **Effective Reads** | **AvgLen (bp)** | **GC (%)** | **Q20 (%)** | **Q30 (%)** | **Effective (%)** |
| --- | --- | --- | --- | --- | --- | --- | --- | --- |
| Con1 | 79841 | 79689 | 78078 | 421 | 54.16 | 99.39 | 97.24 | 97.79 |
| Con2 | 80061 | 79885 | 77322 | 420 | 53.99 | 99.37 | 97.18 | 96.58 |
| Con3 | 79752 | 79596 | 77693 | 420 | 54.12 | 99.36 | 97.15 | 97.42 |
| In1 | 79872 | 79681 | 75989 | 419 | 54.58 | 99.36 | 97.16 | 95.14 |
| In2 | 80132 | 79961 | 76173 | 419 | 54.77 | 99.38 | 97.25 | 95.06 |
| In3 | 79972 | 79806 | 75716 | 419 | 54.62 | 99.37 | 97.19 | 94.68 |

**Abbreviations:** Con means control group; In means infection group.
